# Human Milk Oligosaccharides Mediate the Host–Microbe Interface in a Model Vaginal Community

**DOI:** 10.1021/acsinfecdis.5c00295

**Published:** 2025-06-03

**Authors:** Julie A. Talbert, Sabrina K. Spicer, Shannon D. Manning, Jennifer A. Gaddy, Steven D. Townsend

**Affiliations:** † Department of Chemistry, 5718 Vanderbilt University, Nashville, Tennessee 37240, United States; ‡ Department of Microbiology, Genetics, and Immunology, Michigan State University, East Lansing, Michigan 48824, United States; § Department of Medicine, Vanderbilt University Medical Center, Nashville, Tennessee 37232, United States; ∥ Department of Veterans Affairs, Tennessee Valley Healthcare Systems, Nashville, Tennessee 37212, United States; ⊥ Department of Pathology, Microbiology and Immunology, Vanderbilt University Medical Center, Nashville, Tennessee 37232, United States

**Keywords:** group B Streptococcus, lactobacillus, human milk oligosaccharides, microbiome

## Abstract

Group B Streptococcus (GBS) is an opportunistic bacterium that can cause severe infection during gestation, leading to adverse pregnancy outcomes and neonatal disease. As current treatments only decrease chances of early onset neonatal disease without impacting the risk of chorioamnionitis, preterm birth, or late-onset disease, novel therapeutics are needed. Here, we demonstrate that human milk oligosaccharides (HMOs) positively modulate cocultures of GBS and Lactobacillus spp., common inhabitants of a healthy vaginal microbiome, across in vitro, ex vivo, and in vivo experiments. HMOs shift the total cell population in vitro to favor Lactobacillus, which was qualitatively visualized via scanning electron microscopy. Lactobacillus adherence to EpiVaginal tissues was also increased with HMOs during coinoculation with GBS. Using an in vivo mouse model of reproductive GBS infection, Lactobacillus crispatus and HMOs prevented ascending infection, reducing bacterial burden in both the placenta and fetus. L. crispatus alone reduced the burden in all reproductive tissues tested except the vagina. Together, these results highlight the benefit of pre- and probiotic treatment to potentially reduce GBS colonization during gestation.

## Graphical abstract

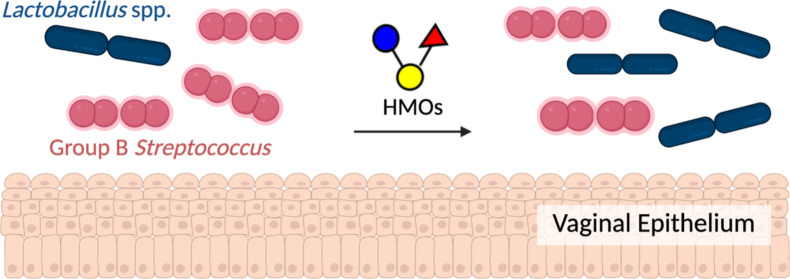

Microbial dysbiosis, an imbalance in the composition of a microbial community, has been recently linked to various health concerns, including cardiovascular disease, cancer, digestive diseases, skin conditions, mental disorders, and many more.^1^ The listed health concerns are normally attributed to dysbiosis of the gut microbiota, but imbalances of other niches, such as the vagina, are also concerning. Most healthy vaginal microbial communities are dominated by Lactobacillus spp., predominantly L. crispatus, L. gasseri, L. jensenii, and L. iners.^2,3^ Vaginal cavities lacking an abundance of Lactobacillus spp. can lead to conditions such as bacterial vaginosis^4,5^ and urinary tract infections,^6,7^ as well as an increased susceptibility to sexually transmitted infections^8^ and pelvic inflammatory disease.^9^ Additionally, dysbiosis increases the risk of habitation by pathogens. An opportunistic pathogen of the vaginal niche is Streptococcus agalactiae (Group B Streptococcus, GBS), which is a common member of the gastrointestinal and urogenital tracts of healthy individuals. However, GBS can cause disease in immunocompromised patients, such as pregnant women and neonates. During gestation, GBS infection can lead to various adverse outcomes, including inflammation of the membrane surrounding the fetus (chorioamnionitis), preterm premature rupture of membranes (PPROM), preterm birth, neonatal and maternal sepsis, and fetal or maternal demise.^10–15^ During pregnancy, GBS can ascend from the vaginal microbiota to infect the developing fetus *in utero*, causing early onset disease in infants. Babies can also acquire GBS through vertical transmission while passing through the vaginal cavity or by horizontal transmission nosocomially.^15–17^

Various studies have demonstrated that vaginal GBS colonization is associated with decreased levels of Lactobacillus spp., specifically L. crispatus.^18,19^ Although lactobacilli acidify the environment through production of lactic acid and hydrogen peroxide, GBS has developed mechanisms to evade some of this acidification.^20^ Current treatment for GBS during pregnancy involves intrapartum antibiotic prophylaxis (IAP),^21^ but recently, concerns have evolved about rising rates of antibiotic resistance and the negative impact of antibiotics on the developing neonatal microbiome.^22,23^ In this context, human milk oligosaccharides (HMOs) have emerged as a promising alternative.

Previously, our group has investigated the effects of HMOs on several pathogens including GBS, methicillin-resistant Staphylococcus aureus (MRSA), and Acinetobacter baumannii.^24–26^ HMOs are the third most abundant macromolecule in human breast milk. Although indigestible by the infant, they act as prebiotics while also defending the neonatal microbiota from pathogenic colonization.^27^ We took an interest in expanding HMO use outside of neonatal health, and our findings demonstrated that these oligosaccharides are potent antimicrobial and antibiofilm agents against varying strains of GBS in vitro.^28^ Further, these activities extend into ex vivo models and an in vivo pregnancy murine model.^29^ With this knowledge in hand, it is also important to examine the effects of HMOs on commensal bacteria inhabiting similar ecological niches. Although it is known that HMOs are beneficial for commensal bacteria, most of this work has focused on gastrointestinal bacteria in soloculture. Herein, we demonstrated a heightened focus on the effects of HMOs on vaginal Lactobacillus spp. alone and in coculture with GBS in vitro but also on vaginal organoids and in an in vivo murine model of ascending infection. By creating a model vaginal community, we can acquire a better understanding of the interactions between GBS and Lactobacillus spp. in the presence of HMOs.

## Results

### HMOs Enhance the Growth of Vaginally Prominent Lactobacillus Strains

As human breast milk contains 5–25 mg/mL HMOs at any point during lactation,^30–32^ we chose to perform growth assays for all strains at 2.5 and 5.0 mg/mL. HMOs for all assays herein were isolated and purified from human breast milk and pooled to create a heterogeneous mixture. Activity was evaluated by comparing growth of Lactobacillus spp. in medium alone to growth in medium with 2.5 or 5.0 mg/mL HMO supplementation (Figure 1A). For L. crispatus 33820, growth was significantly increased compared to medium alone at 24 and 48 h for 2.5 mg/mL HMO (*P* = 0.0049 and *P* = 0.0037, respectively) and 5.0 mg/mL HMO (*P* = 0.0031 and *P* = 0.0039, respectively). For L. crispatus 53545, growth was significantly increased compared to medium alone at 24 and 48 h for 2.5 mg/mL HMO (*P* = 0.0268 and *P* = 0.0016, respectively) and 5.0 mg/mL HMO (*P* = 0.0055 and *P* = 0.0029, *P* = 0.0021, respectively). L. gasseri growth was significantly increased compared to medium alone at 36 h for 2.5 and 5.0 mg/mL HMO (*P* = 0.0003 and *P* = 0.0037, respectively). Notably, the growth cycle of L. gasseri was less than 48 h; thus, its growth and viability curves were shortened to 36 h. L. iners growth was significantly increased compared to medium alone at 12, 16, 24, and 48 h for 2.5 mg/mL HMO (*P* = 0.0125, *P* = 0.0044, *P* = 0.0098, and *P* = 0.0465, respectively) and 5.0 mg/mL HMO (*P* = 0.0153, *P* = 0.0040, *P* = 0.0305, and *P* = 0.0157, respectively). All significance was measured using two-way ANOVA with Dunnett’s post hoc multiple comparisons test (*N* = 3).

**Figure 1:**
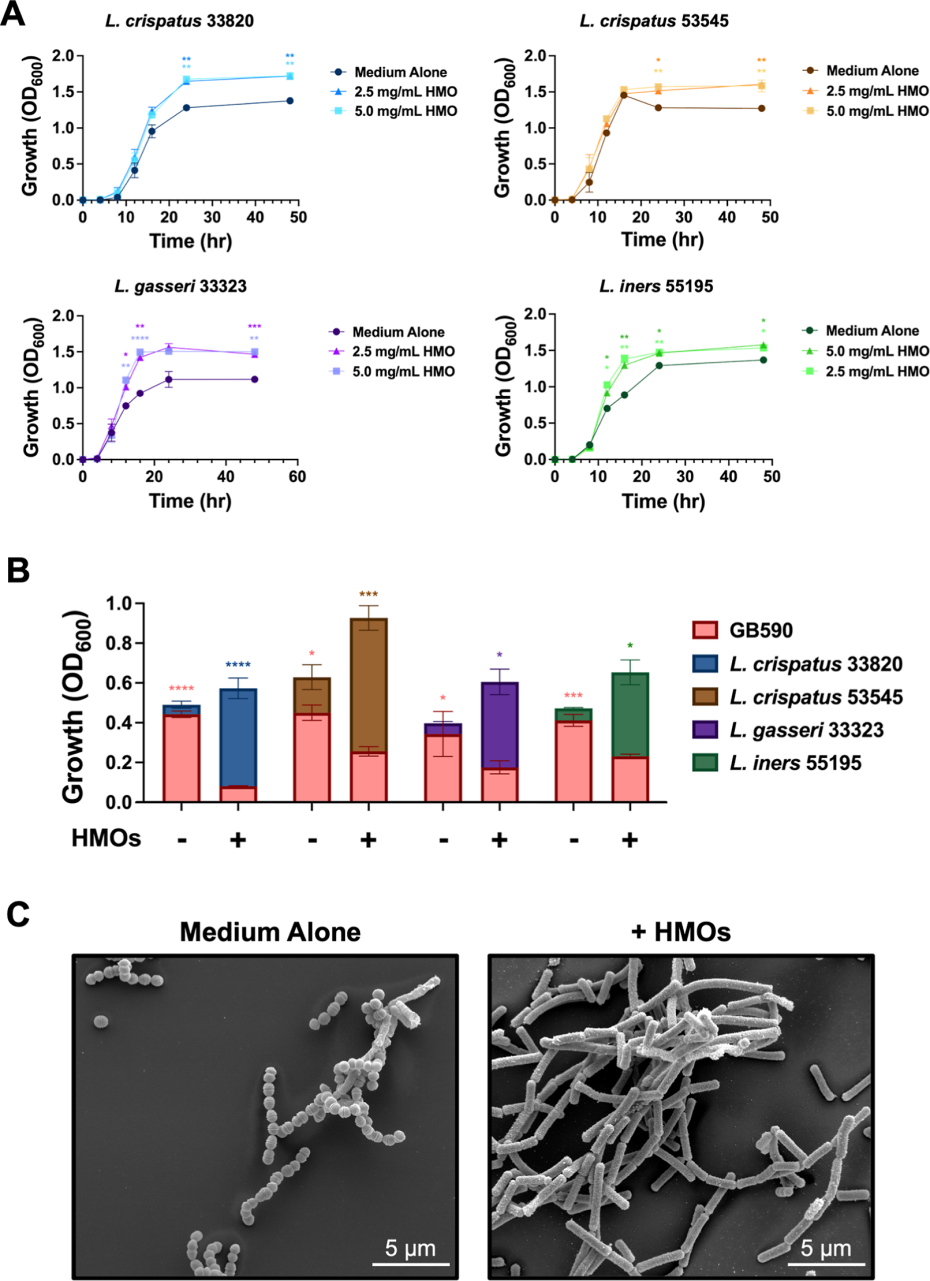
HMOs increase growth of prominent vaginal Lactobacillus strains and positively affect interactions in coculture with GBS. (A) HMOs (2.5 and 5 mg/mL) increase the growth of L. crispatus 33820, L. crispatus 53545, L. gasseri 33323, and L. iners 55195. Symbols indicate mean ± SEM. Star color corresponds to the HMO dose used for the comparison. (B) Quantitative data illustrating that in medium alone, GBS dominates the total cell population of the well. However, HMOs (5 mg/mL) reverse this phenotype, allowing Lactobacillus to dominate. Bars indicate mean ± SEM. Star color indicates which bacterium dominated the cell population. (C) High-resolution field-emission gun-scanning electron microscopy (FEG-SEM) of GB590 and L. crispatus 33820 in medium alone and medium supplemented with HMOs (2.5 mg/mL). Micrographs were collected at 10,000× magnification and are representative of 3 biological replicates. *P < 0.05, **P < 0.01, ***P < 0.001, ****P < 0.0001 by Dunnett’s post hoc test following two-way ANOVA (panel A, comparing medium alone to HMO-supplemented conditions at each time point; *N* = 3) or by one-way ANOVA (panel B, comparing bacterial growth of GBS to Lactobacillus at 24 h; *N* = 3).

### HMOs Only Enhance the Viability of L. Crispatus 33820

We also wanted to investigate the viability of Lactobacillus strains via colony forming unit (CFU) formation in medium alone and medium with HMO supplementation at 2.5 and 5.0 mg/mL (Figure S1). For L. crispatus 33820, viability was significantly increased compared to medium alone at 24 and 48 h for 2.5 mg/mL HMO (*P* = 0.0183 and *P* = 0.0008, respectively) and 5.0 mg/mL HMO (*P* = 0.0175 and *P* = 0.0127, respectively). There was no significant change in the viability of L. crispatus 53545 at any time point. For both L. gasseri and L. iners, there were significant decreases at their final time points for 2.5 mg/mL (*P* = 0.0057 and *P* = 0.0174, respectively). Additionally, 5.0 mg/mL HMO caused a decrease in the viability of L. gasseri at 36 h (*P* = 0.0402). All significance was measured using two-way ANOVA with Dunnett’s post hoc multiple comparisons test (*N* = 3).

### HMOs Positively Mediate Commensal–Pathogen Interaction in a Dual-Species Model

After observing that HMOs increase the growth of four vaginal Lactobacillus strains, we wanted to assess the impact of HMOs on a maternal colonizing strain of GBS (GB590)^33^ and each Lactobacillus strain. To accomplish this, we used transwell inserts containing a semipermeable membrane, which allows communication between GBS and Lactobacillus but prevents direct contact of the bacteria being tested. In the unsupplemented cocultures with L. crispatus 33820, L. crispatus 53545, L. gasseri 33323, and L. iners 55195 with GB590, GBS dominated the total cell population (*P* < 0.0001, *P* = 0.0116, *P* = 0.0391, *P* = 0.0003, respectively, Figure 1B). However, when treated with HMOs at the minimum inhibitory concentration (MIC) of the cocktail used for these experiments (5.0 mg/mL), L. crispatus 33820, L. crispatus 53545, L. gasseri 33323, and L. iners 55195 outcompeted GBS (*P* < 0.0001, *P* = 0.0009, *P* = 0.0457, *P* = 0.0125, respectively, Figure 1B). Notably, HMOs were added to both sides of the semipermeable membrane. All significance was determined via one-way ANOVA with Dunnett’s post hoc multiple comparisons test (*N* = 3). This quantitative data was validated via field-emission gun scanning electron microscopy (FEG-SEM). Growing L. crispatus 33820 and GB590 on glass coverslips in medium alone demonstrated that GBS dominated the coverslip as illustrated by the strong presence of diplococci (Figure 1C). However, HMO supplementation (5.0 mg/mL) on this same coculture reversed the population distribution, where rod-shaped lactobacilli became the predominant cell type (Figure 1C).

### HMOs Shift Cell Population of Lactobacillus and GBS on EpiVaginal Tissues

To investigate HMO action on the cocultures ex vivo, we used a reconstructed EpiVaginal human organoid tissue model, which was qualitatively analyzed via FEG-SEM, as previously described.^29^ First, we examined the impact of HMOs on the adherence of L. crispatus 53545, L. gasseri 33323, L. iners 55195, and GB590 to these vaginal tissues individually. Across all tested strains, HMOs (10 mg/mL) appeared to decrease the adherence of all cells (Figure S2). Notably, 10 mg/mL HMO was used, as this was the MIC of the HMO cocktail used at the time of the ex vivo experiments. Although HMOs decreased the adherence of Lactobacillus, we still wanted to investigate if HMOs can positively modulate the cell population when grown in coculture on these tissues. To test this, L. crispatus 53545, L. gasseri 33323, and L. iners 55195 were each cocultured with GB590 on EpiVaginal tissue. In the unsupplemented cultures, GBS adhered across the entirety of the tissue with a small presence of lactobacilli (Figure 2, top panel). Supplementing the media with HMOs (10 mg/mL) resulted in a greater presence of rod-shaped lactobacilli (Figure 2, bottom panel).

**Figure 2:**
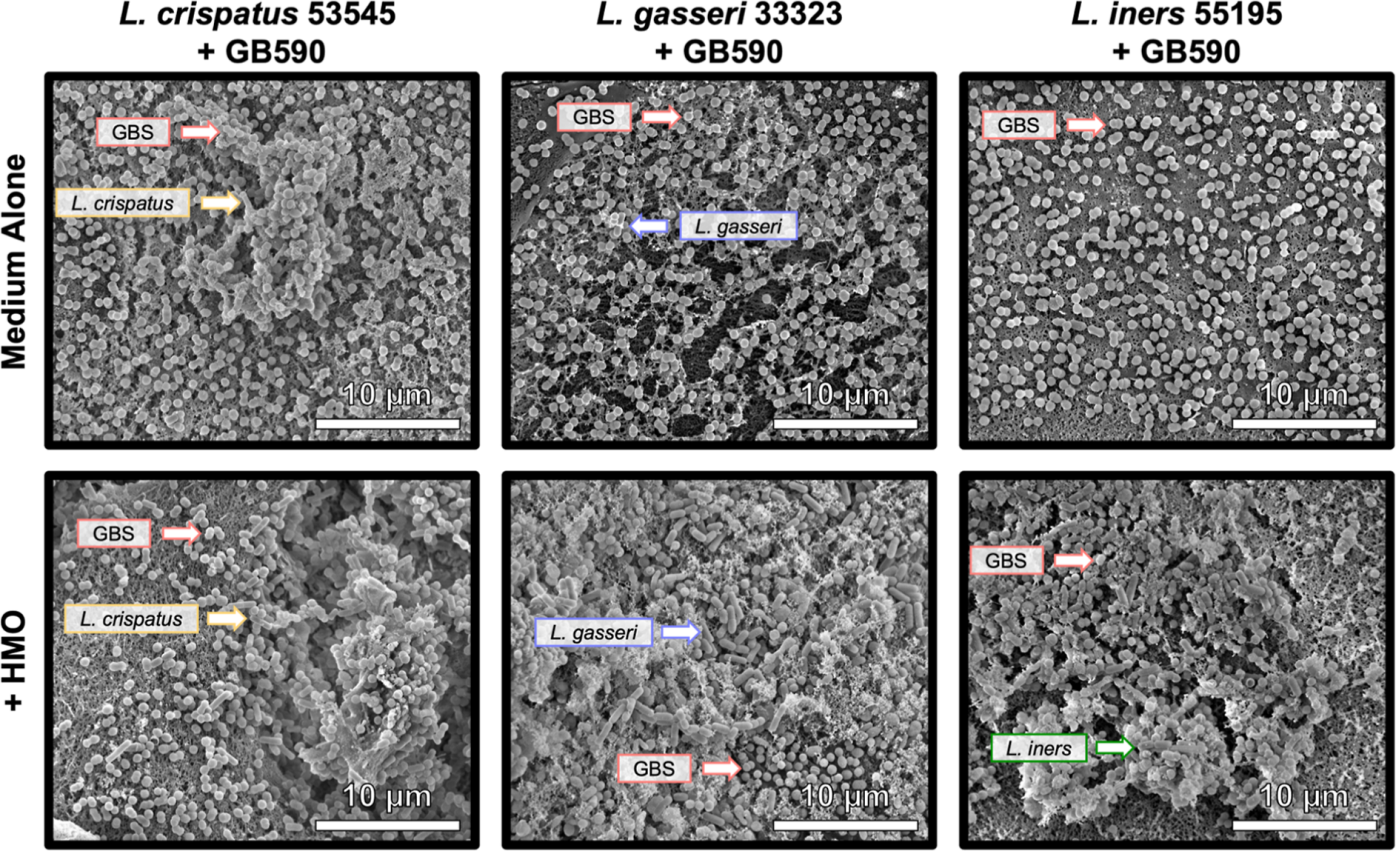
HMOs increase abundance of Lactobacillus on EpiVaginal tissue when cocultured with GBS. High-resolution field-emission gun scanning electron microscopy (FEG-SEM) analyses of GB590 in coculture with L. crispatus 53545, L. gasseri 33323, or L. iners 55195 on vaginal tissue. FEG-SEM imaging of bacterial adherence was performed on bacteria grown with vaginal tissues in medium alone (top) or with 10 mg/mL HMOs (bottom). Micrographs were collected at 10,000× magnification and are representative of 3 biological replicates.

### Lactobacillus Reduces Bacterial Burden during GBS Infection

To investigate the activity of Lactobacillus and HMOs in vivo, we used a murine model of vaginal colonization and ascending GBS infection during gestation.^29,34,35^ Based on our previous data, we hypothesized that the combination treatment of L. crispatus 33820 and HMOs would prevent ascending GBS infection in this pregnant mouse model. For the experimental setup, we wanted to mimic both the EpiVaginal cocultures and the previous murine experiment that demonstrated that HMOs can prevent GBS ascending infection. As such, HMOs were vaginally dosed (10–25 mg/kg) on embryonic day 12.5 (E12.5) (Figure 3A). On E13.5, both GB590 and L. crispatus 33820 were vaginally inoculated at 1 × 10^6^ CFU. Uninfected and untreated controls were also maintained. On E14.5, mice were vaginally treated again with HMOs. All mice were sacrificed 48 h postinfection on E15.5, and necropsy was performed on the vagina, uterus, decidua, placenta, amnion, and fetus (Figure 3B).

**Figure 3:**
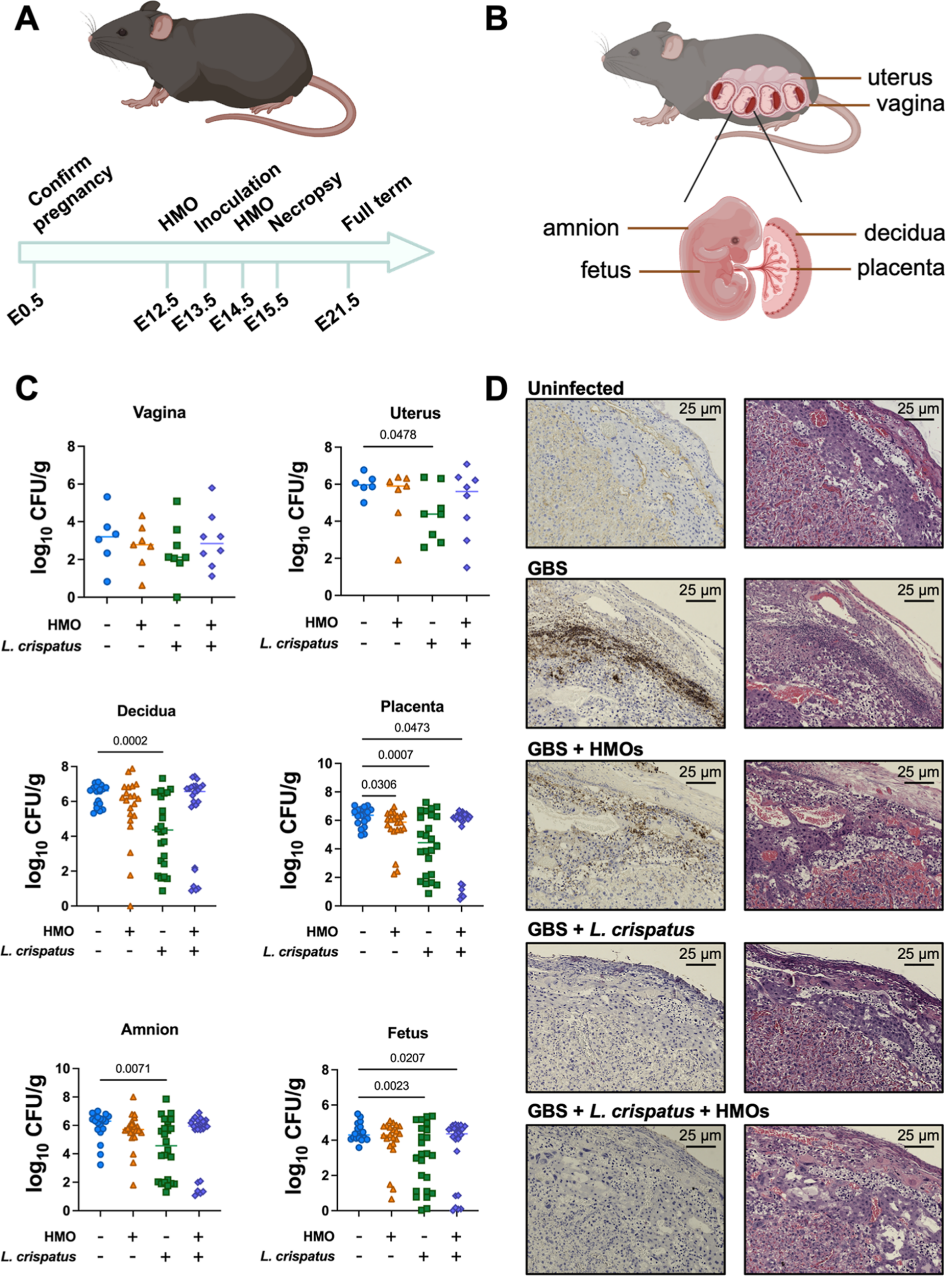
L. crispatus alleviates burden of GBS infection during pregnancy. (A) Conceptual diagram of these studies. Pregnant mice were treated with HMOs on embryonic day 12.5 (E12.5), vaginally inoculated with GBS (GB590) and L. crispatus (ATCC 33820) on E13.5, and treated again with HMOs on E14.5. Mice were sacrificed on E15.5. (B) Diagram of reproductive tissues collected during necropsy. (C) Bacterial burden within reproductive tissues (vagina, uterus, decidua, placenta, amnion, and fetus) evaluated by quantitative culture. Significance calculated via one-way ANOVA with Dunnett’s T3 post hoc multiple comparisons test. *N* = 6–8 dams with 3 tissues analyzed per dam for the decidua, placenta, amnion, and fetus. (D) Placental units analyzed by immunohistochemical techniques (left) using a polyclonal antibody to stain specifically for GBS (indicated by brown stain) and by hematoxylin and eosin staining (right). Micrographs were taken at 10× magnification and are representative of 5 biological replicates.

In the vagina, there was no difference in the bacterial burden across the tested groups (Figure 3C). However, a significant decrease in GBS burden was observed with L. crispatus treatment in uterine, decidual, and amnion tissues (*P* = 0.0478, *P* = 0.0002, *P* = 0.0071, respectively). For the placental tissue, HMOs, L. crispatus, and the combination of the two resulted in a significant decrease in bacterial burden (*P* = 0.0306, *P* = 0.0007, *P* = 0.0473, respectively). Finally, L. crispatus alone and in combination with HMOs also significantly decreased the bacterial burden in the fetus (*P* = 0.0023, *P* = 0.0207, respectively). Although the combination treatment did not significantly reduce burden in the decidua and amnion, the data show decreasing trends (*P* = 0.1443 and *P* = 0.1719, respectively). All bacterial burden analyses were calculated via one-way ANOVA with Dunnett’s T3 post hoc multiple comparisons test. *N* = 6–8 dams with 3 tissues analyzed per dam for the decidua, placenta, amnion, and fetus.

### Lactobacillus Alone and in Combination with HMOs Reduces Inflammation of Reproductive Tissues

Previously, we have demonstrated that GBS causes placental inflammation of the reproductive tract of pregnant mice.^34,36^ To determine whether HMOs or Lactobacillus can influence this inflammation, we deployed immunohistochemical (IHC) and histopathological staining on the tested tissues. IHC staining, using an anti-GBS lysate, demonstrated that GB590 infiltrated the fetal-placenta units, as indicated by the brown strain (Figure 3D, left panel). Gratifyingly, L. crispatus 33820 alone and in combination with HMOs decreased the GBS invasion, comparable to the uninfected tissue. HMOs also slightly decreased the level of GBS infection in these tissues. The hematoxylin and eosin staining illustrated that GBS infection increased inflammation, as depicted by the polymorphonuclear cell infiltration and minor loss of tissue integrity (Figure 3D, right panel). HMOs, L. crispatus, and the combination of both decreased the polymorphonuclear cell permeation and tissue disruption compared to that of GBS alone.

### Lactobacillus Reverses Proinflammatory Cytokine/Chemokine Response to GBS Infection

As the murine model data illustrate that Lactobacillus reduces bacterial burden across most tissues and reduces burden in the placenta and fetus in combination with HMOs, we hypothesized that this could be associated with positive modulation of the immune response. To test this, we used multiplex cytokine assays to determine the levels of proinflammatory cytokines and chemokines in five groups: uninfected, infected (GB590), infected and HMO-treated, infected and L. crispatus 33820 treated, and infected and L. crispatus 33820 plus HMO treated. Across the tissues, we found that GBS infection significantly increases the level of proinflammatory cytokines and chemokines (Figures 4–7, S3–6, and Table S1). Notably, Lactobacillus alone reduced proinflammatory cytokine/chemokine levels more than HMOs alone or with the combination treatment. All cytokine analysis was performed using a Student’s *t*-test, *N* = 3.

**Figure 4:**
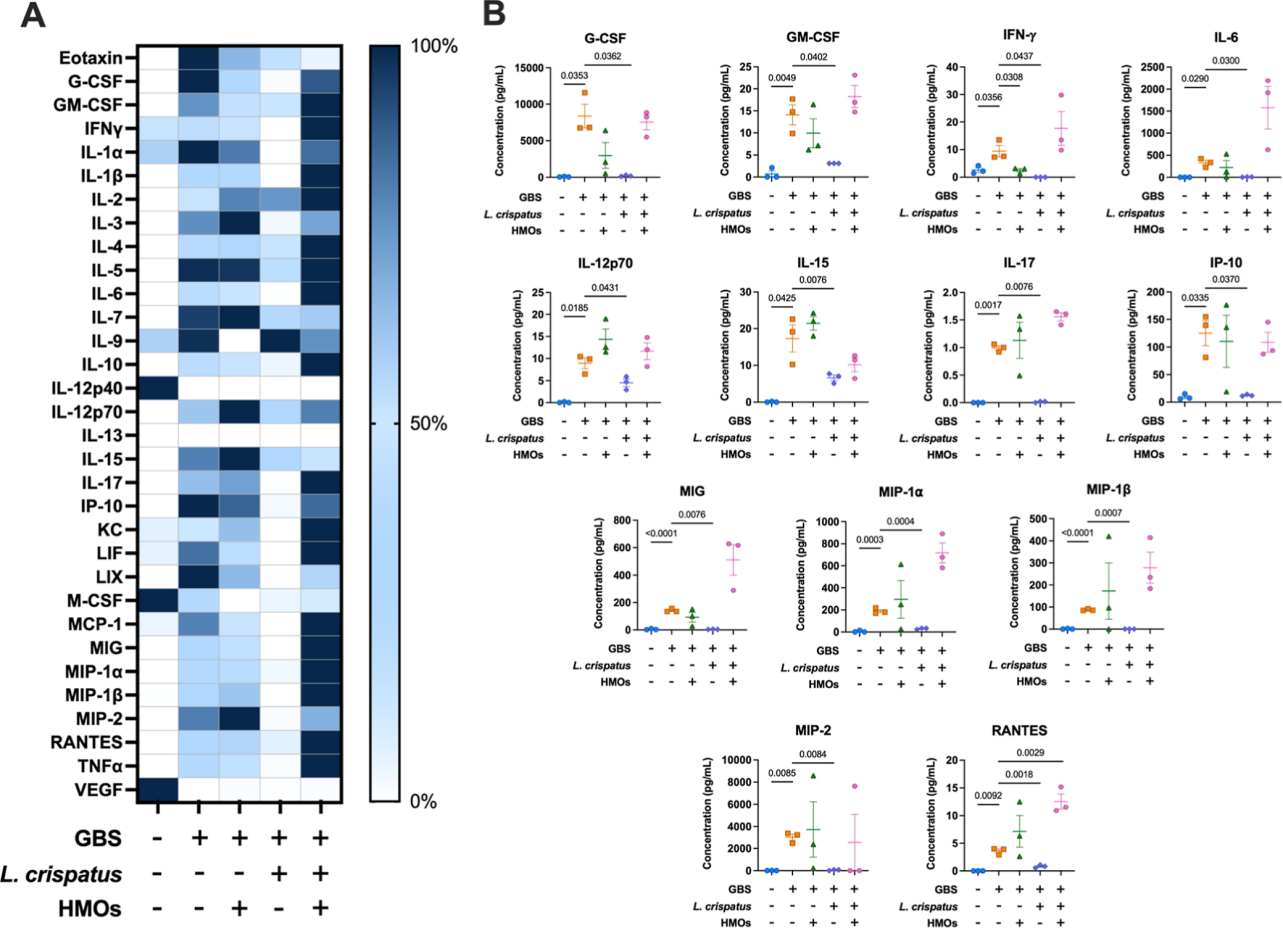
Analysis of cytokine and chemokine production in decidual tissue in response to GBS infection with HMO, L. crispatus, or HMO + L. crispatus treatment. (A) Heat map visualization of normalized cytokine levels. Dark blue = high expression and white = low expression. (B) Quantification of significantly affected proinflammatory cytokines/chemokines via multiplex analyses of decidual tissue after ascending vaginal infection with no infection or treatment (blue circles), GB590 (orange squares), GB590 with HMOs (green triangles), GB590 with L. crispatus 33820 (purple diamonds), and GB590 with HMOs and L. crispatus 33820 (pink hexagons). Decidual tissues were collected from pregnant dams on E15.5, 2 days post inoculation. Errors bars represent the standard error mean with individual data points representing analysis of tissue from separate dams. Significance determined via Student’s *t*-test, *N* = 3. *P* values < 0.05 were considered significant and those resulting *p* values are listed on the individual cytokine graphs.

G-CSF, GM-CSF, and RANTES levels increased across all tissues with GBS infection compared with uninfected controls. Lactobacillus treatment, though, significantly decreased the production of these cytokines and chemokines relative to the infected tissues. Similarly, GBS infection increased the level of IFN-γ, IP-10, MIP-1α, MIP-1β, and MIP-2 in the decidua, placenta, and amnion, with Lactobacillus addition significantly decreasing these levels. Similarly, IL-15 and MIG exhibited increased production with GBS infection in the decidua and placenta, while Lactobacillus reduced their production compared to GBS alone. IL-1β and LIX illustrated enhanced production with GBS, while Lactobacillus again restored these levels. In general, the observed trend was that GBS infection significantly increased production of proinflammatory cytokines/chemokines, including G-CSF, GM-CSF, INF-γ, IL-6, IL-12p70, IL-15, IL-17, IP-10, MIG, MIP-1α, MIP-1β, MIP-2, and RANTES in the decidua (Figures 4, S3), G-CSF, GM-CSF, INF-γ, IL-1α, IL-1β, IL-12p70, IL-15, IL-17, IP-10, LIX, MCS-F, MIG, MIP-1α, MIP-1β, MIP-2, and RANTES in the placenta (Figures 5, S4), G-CSF, GM-CSF, INF-γ, IL-1α, IP-10, MCP-1, MCS-F, MIP-1α, MIP-1β, MIP-2, RANTES, and TNF-α in the amnion (Figure 6, S5), and G-CSF, GM-CSF, IL-1α, IL-1β, IL-12p70, IL-17, LIX, RANTES, and TNF-α in the fetus (Figures 7, and S6). For all mentioned cytokines/chemokines, Lactobacillus treatment significantly reduced their production compared to GBS infection (Table S1).

**Figure 5:**
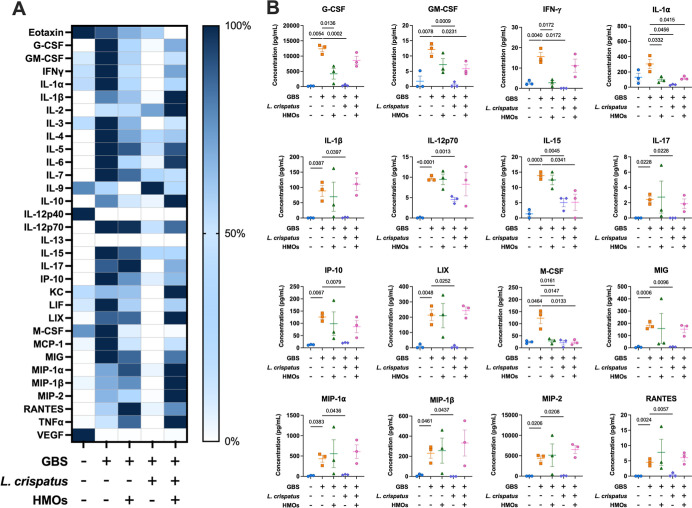
Analysis of cytokine and chemokine production in placental tissue in response to GBS infection with HMO, L. crispatus, or HMO + L. crispatus treatment. (A) Heat map visualization of normalized cytokine levels. Dark blue = high expression and white = low expression. (B) Quantification of significantly affected proinflammatory cytokines/chemokines via multiplex analyses of placental tissue after ascending vaginal infection with no infection or treatment (blue circles), GB590 (orange squares), GB590 with HMOs (green triangles), GB590 with L. crispatus 33820 (purple diamonds), and GB590 with HMOs and L. crispatus 33820 (pink hexagons). Placental tissues were collected from pregnant dams on E15.5, 2 days post inoculation. Errors bars represent the standard error mean with individual data points representing analysis of tissue from separate dams. Significance determined via Student’s *t*-test, *N* = 3. *P* values < 0.05 were considered significant and those resulting *p* values are listed on the individual cytokine graphs.

**Figure 6:**
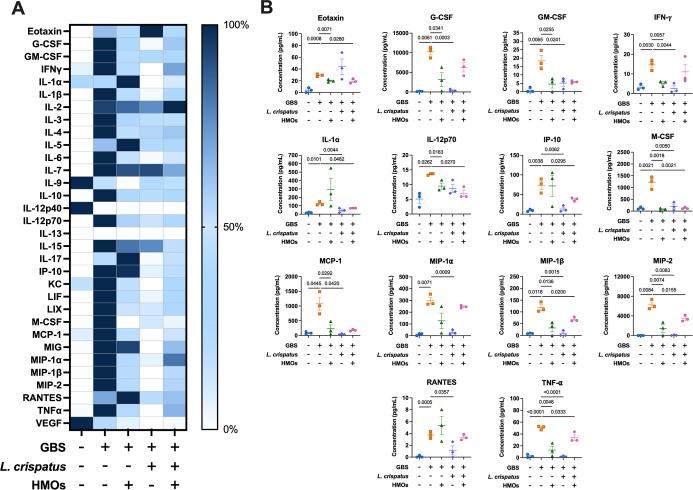
Analysis of cytokine and chemokine production in amnion tissue in response to GBS infection with HMO, L. crispatus, or HMO + L. crispatus treatment. (A) Heat map visualization of normalized cytokine levels. Dark blue = high expression and white = low expression. (B) Quantification of significantly affected proinflammatory cytokines/chemokines via multiplex analyses of amnion tissue after ascending vaginal infection with no infection or treatment (blue circles), GB590 (orange squares), GB590 with HMOs (green triangles), GB590 with L. crispatus 33820 (purple diamonds), and GB590 with HMOs and L. crispatus 33820 (pink hexagons). Amnion tissues were collected from pregnant dams on E15.5, 2 days post inoculation. Errors bars represent the standard error mean with individual data points representing analysis of tissue from separate dams. Significance determined via Student’s *t*-test, *N* = 3. *P* values < 0.05 were considered significant and those resulting *p* values are listed on the individual cytokine graphs.

**Figure 7:**
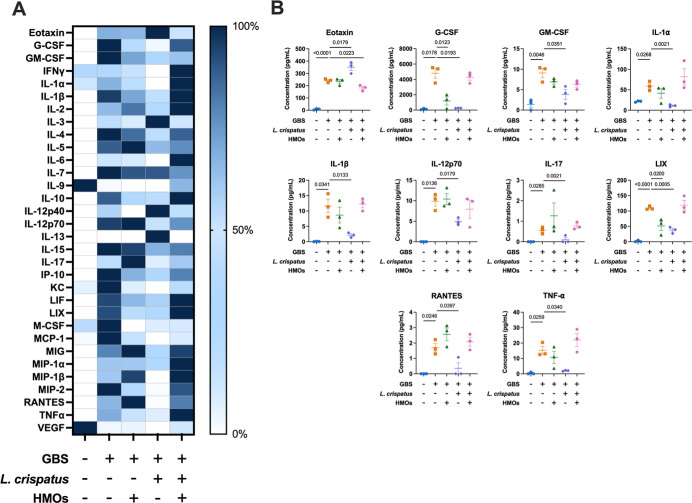
Analysis of cytokine and chemokine production in fetal tissue in response to GBS infection with HMO, L. crispatus, or HMO + L. crispatus treatment. (A) Heat map visualization of normalized cytokine levels. Dark blue = high expression and white = low expression. (B) Quantification of significantly affected proinflammatory cytokines/chemokines via multiplex analyses of fetal tissue after ascending vaginal infection with no infection or treatment (blue circles), GB590 (orange squares), GB590 with HMOs (green triangles), GB590 with L. crispatus 33820 (purple diamonds), and GB590 with HMOs and L. crispatus 33820 (pink hexagons). Fetal tissues were collected from pregnant dams on E15.5, 2 days post inoculation. Errors bars represent the standard error mean with individual data points representing analysis of tissue from separate dams. Significance determined via Student’s *t*-test, *N* = 3. *P* values < 0.05 were considered significant, and those resulting *p* values are listed on the individual cytokine graphs.

For most pro-inflammatory markers, combination treatment of Lactobacillus and HMOs did not reduce the level of cytokine activity. The only reduction in proinflammatory cytokine levels with the combination treatment was eotaxin, IL-12p70, and MCS-F in the amnion (Figure 6) and eotaxin and MCS-F in the fetus (Figure 7). HMOs alone reduced proinflammatory cytokines/chemokines including INF-γ and MCS-F in the decidua (Figures 4, S3), G-CSF, INF-γ, IL-1α, and MCS-F in the placenta (Figure 5), eotaxin, G-CSF, GM-CSF, INF-γ, IL-12p70, MCP-1, MCS-F, MIP-1β, MIP-2, and TNF-α in the amnion (Figure 6), and G-CSF, LIX, and MCS-F in the fetus (Figures 7, S6).

## Discussion

GBS is an opportunistic pathogen that presents a high risk to immunocompromised patients such as pregnant individuals and neonates. Even with current treatment protocols in place, GBS disease remains a leading cause of infant morbidity and mortality.^15,37^ As such, novel therapies are needed to combat this type of bacterial infection. Our group has investigated HMOs as antibiofilm and antimicrobial agents, with the first study illustrating HMO activity against GBS in 2017.^38^ Expansion of this study included HMO antibiofilm activity against MRSA and A. baumannii.^25,26^ Since the impetus of this project, we have published extensively on HMOs spanning commentary on viral infections to HMOs as antibiotic adjuvants to our most recent study highlighting HMOs in their ability to prevent ascending GBS infection in the murine mouse model.^29,39,40^ Across these reports, we have noticed a need to better represent the vaginal microbiome, as this is the primary niche in which GBS causes infection. In this vein, we expanded the study to include Lactobacillus species that are commonly found in a healthy vaginal microbiota and have previously been shown to interact with both GBS and placental membranes.^41,42^

Herein, we demonstrated for the first time that the growth of vaginal Lactobacillus strains is significantly increased with HMO supplementation. The viability of L. crispatus 33820 also increased with HMOs; however, L. crispatus 53545, L. gasseri 33323, and L. iners 55195 did not exhibit similar results, with their viabilities either decreasing or remaining unchanged. These intriguing results warrant further mechanistic experimentation into how HMOs can simultaneously increase the growth of all tested Lactobacillus strains yet affect viabilities variably.

Most studies investigating HMO metabolism in lactobacilli have used the primarily gastrointestinal strains, such as L. reuteri, L. acidophilus, and L. rhamnosus.^43–46^ Generally, the gut-residing strains are unable to metabolize HMOs, except lacto-*N*-neotetraose (LNnT), which is attributed to their β-galactosidase activity. These studies often include the gastrointestinal strain as HMOs from breast milk are greeted by members of bifidobacteria and lactobacilli in the neonatal gut. However, since bifidobacteria remain capable of digesting these complex oligosaccharides,^43,46^ we postulate that, evolutionarily, gut Lactobacillus species either lost or ceased expressing the genes required for HMO metabolism. In the vaginal microbiome, however, lactobacilli often dominate healthy niches, with limited counterparts to digest such sugars. Here, we have shown that HMOs cause increased growth of L. crispatus, L. gasseri, and L. iners, alluding to their retention of the machinery required to break down HMOs for use as an energy source.

Although L. crispatus, L. gasseri, L. jensenii, and L. iners are among dominant members of a healthy vaginal microbial community,^2,47^ there is evidence suggesting that shifting toward a heavy L. iners population can lead to dysbiosis, as reviewed by Ling and colleagues.^48^ As dysbiosis is associated with various diseases and infections, an L. iners-dominated microbiome is undesirable. Yet, as HMOs promote growth across each tested Lactobacillus strain, we see these data in a positive light.

With previous evidence that HMOs inhibit GBS growth,^25^ we tested HMOs in a coculture of Lactobacillus and GBS to reflect microbial interactions more accurately in the vaginal microbiome. Various studies have demonstrated a direct antagonism between lactobacilli and GBS in assays where cells interact directly.^41,49,50^ In light of this, we employed transwells, cell culture inserts with a semipermeable membrane, allowing bacterial communication without physical contact to illustrate how HMOs modulate the indirect interaction between species. The addition of the semipermeable barrier in the cocultures allowed GBS to outcompete Lactobacillus, illustrating that the unsupplemented inhibitory activity of Lactobacillus may require cell-to-cell contact. Gratifyingly, HMOs shift the total cell population of the culture to favor Lactobacillus across all strains tested despite the timing of these assays, which were conducted in a 24 h time frame, favoring GBS growth as Lactobacillus has a 36–48 h cell cycle. Additionally, the bacteria were cultured in Todd Hewitt Broth (THB), a medium that primarily supports GBS growth over Lactobacillus. Despite putting Lactobacillus at a stark disadvantage, we demonstrate that HMO supplementation promotes lactobacilli growth, which may enhance its ability to outcompete GBS.

Mechanistically, we envision two potential reasons that HMOs cause this shift in the cell population. First, as the transwells are semipermeable, HMOs on each respective side could be increasing the growth of Lactobacillus, while simultaneously stunting the growth of GBS. Alternatively, HMOs could be enhancing the chemical warfare of Lactobacillus, increasing production of metabolites that inhibit streptococcal growth. Indeed, a prior collaborative study found that supernatants from various Lactobacillus strains, including L. gasseri 33323, used herein, inhibit the growth of GBS.^41^ Future work in this area will investigate any increased production of metabolites in the supernatant of Lactobacillus that inhibit the growth of GBS.

Because GBS infections originate vaginally, we extended the in vitro experimentation to ex vivo EpiVaginal organoid tissues. HMOs acted as antiadhesives in the solocultures, preventing the adherence of both GBS and Lactobacillus, agreeing with our previous report that illustrated diminished adherence of GBS to vaginal tissues with HMOs.^29^ Cocultures of GBS and Lactobacillus on EpiVaginal tissues demonstrated a complete dominance of GBS across the entirety of the tissue in medium alone akin to previous in vitro data. The addition of HMOs, however, shifted the population, allowing more Lactobacillus cells to adhere to the tissue. While a complete reversal of bacterial dominance was not seen here as in the in vitro assays, the stark increase of rod-shaped lactobacilli on the tissues is promising. As with the data generated in vitro, the experimental setup favored GBS with a 24 h time course and specific EpiVaginal tissue media (VEC-100-MM) that favors GBS growth over Lactobacillus. Together, these findings suggest that HMOs give Lactobacillus an edge to compete for space in the vaginal organoids.

Moving into the murine model of ascending GBS infection, we found that treating with L. crispatus and HMOs significantly decreased the GBS burden in the placenta and fetus. Further, Lactobacillus administration alone significantly reduced the bacterial burden in all reproductive tissues except the vagina. These data match other studies investigating Lactobacillus supplementation in combating GBS infection^51^ and corroborate reports showing that GBS abundance is negatively correlated with Lactobacillus abundance.^19,52,53^ Lactobacillus inoculation also significantly reduced proinflammatory cytokines and chemokines across the decidua, placenta, amnion, and fetus. Specifically, G-CSF, GM-CSF, and RANTES exhibited decreased production in each tissue, while IFN-γ, IL-1α, IL-12p70, IL-17, IP-10, MIP-1α, MIP-1β, and MIP-2 were downregulated in at least three of the tested tissues. As varying studies have associated increased levels of both MIP-1α and MIP-2 with a higher risk of PPROM and preterm birth,^54–56^ we postulate that Lactobacillus intervention could provide a reliable way to both decrease bacterial burden and inhibit this proinflammatory response. Additional studies, however, are required to evaluate the impact more completely on the actual microbiome and cell to cell interactions.

In our previous report, we demonstrated that HMOs significantly reduced the bacterial burden of GBS across murine reproductive.^29^ While the current study was aimed to investigate the HMO impact on the vaginal microbiome, it is important to note that the mouse colony used herein showed an enhanced ability to clear GBS infection. This required a bacterial inoculation ∼1000× more potent than the previous report to achieve a similar GBS disease phenotype. The concentrations of HMOs administered, however, remained consistent between the two independent studies. In the present study, the fetus was the only tissue with diminished bacterial burden in response to HMO treatment; however, we attribute this to the increased bacterial inoculum used.

A final limitation of this study involves the timing of HMO and Lactobacillus treatment compared to GBS inoculation. Here, HMOs were dosed 24 h before and after a coinoculation of GBS and L. crispatus to maintain experimental consistency across in vitro and ex vivo methods. Next steps include altering treatment time frames by establishing either a GBS infection or a protective Lactobacillus environment first and dosing HMOs accordingly. These results will be provided in due course. While preliminary, partial prevention of ascending GBS infection by L. crispatus and HMOs and complete prevention by L. crispatus mark a notable path forward in combatting GBS infection during gestation. Future investigations including vaginal microbiome sequencing will determine how the microbial community changes in response to infection and treatment to enhance our understanding of beneficial and harmful alterations that impact colonization potential and subsequent disease risk.

## Supplementary Material



## Notes

Ethics statement, Institutional review board information, bacterial strains, culture conditions, HMO isolation, bacterial growth and viability assays, coculture assays, epi vaginal coculture, high-resolution field-emission scanning electron microscopy (FEG-SEM) analyses, mouse model of ascending vaginal GBS infection during pregnancy, bacterial burden analysis, histopathological analysis, immunohistochemical analysis, cytokine analysis, and statistical analysis (PDF)
