# Supplementary material for: Human Milk Oligosaccharides Mediate the Host–Microbe Interface in a Model Vaginal Community
Source: ACS Infect Dis. 2025 Jun 3;11(6):1729–40. doi: 10.1021/acsinfecdis.5c00295 (PMC12172043; doi:10.1021/acsinfecdis.5c00295)
Supplement: Supplementary file 1 [file id5c00295_si_001.pdf]

Human Milk Oligosaccharides Mediate the Host-Microbe Interface in a Model Vaginal Community

Julie A. Talbert[1], Sabrina K. Spicer[1], Shannon D. Manning[2], Jennifer A. Gaddy[3,4,5]*, and Steven D. Townsend[1,]*

[1]Department of Chemistry, Vanderbilt University, Nashville, Tennessee 37240, United States

[2]Department of Microbiology, Genetics, and Immunology, Michigan State University, East Lansing, Michigan 48824, United States

[3]Department of Medicine, Vanderbilt University Medical Center, Nashville, Tennessee 37232, United States

[4]Department of Veterans Affairs, Tennessee Valley Healthcare Systems, Nashville, Tennessee 37212, United States

[5]Department of Pathology, Microbiology and Immunology, Vanderbilt University Medical Center, Nashville, Tennessee 37232, United States

[*]Denotes Co-corresponding Authorship

Keywords: Group B *Streptococcus*, *Lactobacillus*, human milk oligosaccharides, microbiome

Materials and Methods

Bacterial Strains and Culture Conditions. *Lactobacillus* spp. were chosen to represent species that were relevant to a healthy vaginal tract (*L. crispatus*, *L. gasseri*, *L. iners*). The specific strains included *L. crispatus* (ATCC 53545) and *L. gasseri* (ATCC 33323), which were used in a prior study,[1] as well as *L. crispatus* (ATCC 33820) and *L. iners* (ATCC 55195). All *Lactobacillus* spp. were grown on tryptic soy agar plates supplemented with 5% sheep blood (blood agar plates) at 37 °C for 48 hr. *L. iners* was cultured in de Man, Rogosa and Sharpe (MRS) supplemented with 4 mM L-cysteine and 1.1 mM of L-glutamine in a GASPAK,[2] while all other *Lactobacillus* spp. were grown in MRS in ambient air or 5% CO_2. For comparison, a previously characterized group B *Streptococcus* (GBS) strain, GB00590 (GB590), was used. This strain was recovered from the vaginal/rectal swab of a colonized pregnant woman and was classified as multilocus sequence type 19 with a serotype III polysaccharide capsule.[3] GBS was grown on blood agar plates and in Todd-Hewitt broth (THB) at 37 °C in ambient air. Following incubation, bacterial density was quantified through absorbance readings at 600 nm (OD_{600}) and bacterial numbers were estimated using the predetermined coefficient of 1 $OD_{600} = 10^9$ colony forming unit (CFU)/mL.

Human Milk Oligosaccharide Isolation. Human breast milk was obtained from five healthy, lactating women between 3 days and 3 months postpartum and stored at −20 °C. The deidentified milk samples were provided by Dr. J. Hendrik Weitkamp from the Vanderbilt University Medical Center Department of Pediatrics under a collection protocol approved by the Vanderbilt University Institutional Review Board (IRB #100897). Milk samples were thawed and centrifuged at 3750 rpm for 45 min. Subsequently, lipid components were removed by skimming. The proteins were removed by diluting with 1:1 v/v absolute ethanol at 4 °C and centrifuging at 3750 rpm for 45 min. The HMO-containing supernatant was concentrated *in vacuo*, dissolved in phosphate buffer (pH 6.5, 0.2 M), and heated to 37 °C. *β*-galactosidase from *Kluyveromyces lactis* was added, and the reaction mixture was stirred until deemed complete by thin layer chromatography (TLC). The reaction mixture was diluted with ethanol at 4 °C, centrifuged, and concentrated *in vacuo*. The remaining glucose and galactose were separated from the HMOs using size exclusion chromatography using P-2 Gel (H2O eluent). The HMOs were dried by lyophilization. Stocks of HMOs at a final concentration of 102.6 mg/mL were aliquoted into 1 mL tubes and frozen at −20 °C until use in biological assays.

Bacterial Growth and Viability Assays. Bacterial strains were grown overnight as described above and used to inoculate fresh media or media supplemented with ca. 2.5 mg/mL or 5.0 mg/mL HMO. Inoculation was completed at a multiplicity of infection (MOI) of 10^5 CFUs per 100 µL of growth media in 96 well tissue culture-treated, sterile polystyrene plates (Corning Inc.). All cultures were grown under static conditions at 37 °C in ambient air or 5% CO_2. Growth was quantified at 0, 4, 8, 12, 24, and 48 hr through spectrophotometric readings at OD_{600}. The final time-point for *L. gasseri* was 36 hr. Viability was assessed through serial dilution and plating onto blood agar plates followed by quantification of viable CFU/mL.

Coculture Assay. Cocultures were set up as previously described.[4] Briefly, bacterial strains were grown overnight as described above and used to inoculate fresh media to achieve 1×10^6 CFU/mL. To 24-well tissue culture-treated, sterile polystyrene plates was added 500 µL of inoculated THB media above and below the semi-permeable membrane. HMOs were added to each side of the semi-permeable membrane to achieve a final concentration of ca. 5.0 mg/mL. Bacteria grown in THB in the absence of HMOs served as the control. Cultures were grown under static conditions at 37 °C in ambient air or in a 5% CO_2 incubator for 24 h. Growth was quantified through spectrophotometric reading at OD_{600}.

Epi Vaginal Coculture. EpiVaginal™ (VEC-100™) tissues were purchased from MatTek Life Sciences. These tissues were cultured from normal, primary human-derived vaginal epithelial cells. The tissues were processed into 8-mm diameter sections, which were placed in Nunc™ single well tissue culture plate inserts containing DMEM medium, F12 medium, and phenol-red cell culture medium supplemented with 5 µg/ml gentamicin (10% of normal gentamicin level) and 0.25 µg/ml amphotericin B. Sections were thawed and incubated overnight at 37 °C in ambient air containing 5% CO_2, washed 3 times with prewarmed, sterile

phosphate buffered saline (pH 7.4), and placed in VEC-100-MM™ (MatTek Life Sciences) maintenance medium. GBS, *L. crispatus* 53545, *L. gasseri* 33323, *L. iners* 55195 or a normalized coculture of species were added to the top face of the tissue at a MOI of 1×10^7 cells per 8-mm diameter membrane, using a predetermined coefficient of bacterial density of 1 $OD_{600} = 10^9$ CFU/mL. HMOs were added to achieve a final carbohydrate concentration of ca. 10.0 mg/mL. Uninfected vaginal tissue samples were also maintained in medium alone and medium supplemented with ca. 10.0 mg/mL HMO. Tissues were incubated at 37 °C in ambient air containing 5% CO_2 for 24 hr and cells were fixed with 2.0% paraformaldehyde and 2.5% glutaraldehyde in 0.05 M sodium cacodylate buffer (Electron Microscopy Sciences, Hatfield, PA) for 24 hr prior to processing for microscopy.

High-Resolution Field-Emission Scanning Electron Microscopy (FEG-SEM) Analyses. Bacterial adherence was analyzed by FEG-SEM as previously described.[5, 6] Briefly, bacteria were cultured in either MRS or THB in wells containing 12 mm glass coverslips coated with poly-L-lysine (Corning, Bedford MA) at 37 °C in ambient air for 24 h. For in vitro, *L. crispatus* 33820 and GB590 were added at a normalized concentration, reaching 1×10^6 CFU/mL per 12 mm glass coverslip. HMOs were added to achieve a final carbohydrate concentration of ca. 2.5 mg/mL. Untreated samples were also maintained. EpiVaginal tissue experiments were completed as described above. Planktonic cells were removed by decanting the culture and washing the wells gently with 1X PBS. Samples were fixed with 2.5% glutaraldehyde and 2.0% paraformaldehyde in 0.05 M sodium cacodylate buffer (pH 7.4, Electron Microscopy Sciences, Hatfield, PA) for 24 hr prior to dehydration by sequential washing with increasing concentrations of ethanol. After dehydration, samples were dried at the critical point with a Tousimis critical point dryer machine, mounted onto aluminum stubs, and then coated with ca. 20 nm of gold by plasma sputter coating. Subsequently, sample edges were painted with colloidal silver (Electron Microscopy Sciences) to facilitate the dissipation of charge from the sample surface. Samples were imaged with an FEI Quanta 250 field-emission gun scanning electron microscope with an accelerating voltage of 5.0 keV, a spot size of 2.5, and a working distance of 10 mm.

Mouse Model of Ascending Vaginal GBS infection During Pregnancy. GBS infection of pregnant mice and subsequent analyses were performed as previously described.[5, 7] Briefly, C57BL6/J mice were purchased from Jackson laboratories and mated in harem breeding strategies (1 male to 3-4 females) overnight. The following day, pregnancy was confirmed by the presence of a vaginal mucus plug establishing the embryonic day (E0.5). On E12.5, pregnant dams were anesthetized via inhalation of isoflurane and vaginally dosed with an HMOs at 10-25 mg/kg. On E13.5, pregnant dams were anesthetized via inhalation of isoflurane and vaginally infected by pipetting 1×10^6 CFU in 0.05 mL of THB plus 10% gelatin directly into the vagina. For co-inoculation, each bacterium was dosed at 1×10^6 CFU in 0.05 mL for a final concentration of 2×10^6 CFU in 0.05 mL. It is important to note that the release of the liquid might create a pressure effect where some of the inocula could be propelled into the cervix. Uninfected controls were also maintained. On E14.5, pregnant dams were anesthetized via inhalation of isoflurane and vaginally dosed with the same HMO cocktail from day E12.5 at the same concentration. On E15.5, animals were euthanized by CO_2 asphyxiation and necropsy was performed to harvest reproductive tissues, including the vagina, uterus, placenta, decidua, amnion, and fetus. Group sizes equaled 6-8 dams with 3 of each tissue (except the vagina and uterus) analyzed per dam.

Quantifying Bacterial Burden in Host Tissues. To determine bacterial burden in reproductive tissues, quantitative culture methods were employed as previously described.[5, 7] Briefly, reproductive tissues were weighed and placed in sterile THB, which is optimized for growth of streptococcal species. Tissues were homogenized and subjected to serial dilution and plating onto blood agar to enumerate bacteria (CFU/mg) in host tissue.

Histopathological Analyses. Reproductive tissues were subjected to a primary fixation in 4% formalin (neutral buffered) overnight. The following day, tissues were embedded in paraffin and sectioned into 5 μm

thick sections for staining and microscopy. Sections were stained with hematoxylin and eosin for histopathological examination and imaged with an EVOS M7000 Imaging System.

Immunohistochemical Analyses. Tissues were fixed in 4% neutral buffered formaldehyde overnight before being embedded into paraffin blocks. Samples were cut into 5 μm sections, and multiple sections were placed on each slide for analysis. Samples were deparaffinized with xylene, and heat-induced antigen retrieval was performed on the Bond Max automated IHC stainer (Leica Biosystems) using Epitope Retrieval 2 solution for 5 to 20 min. Slides were incubated with a rabbit polyclonal antiGBS antibody (ab78846; Abcam) for 1 hr. The Bond Polymer Refine detection system (Leica Biosystems) was used for visualization. Slides were counter stained with eosin, dehydrated and cleared, and coverslips were added before light microscopy analysis was performed.

Cytokine Analyses. The reproductive tissues were analyzed by multiplex cytokine assays. Mouse tissues were placed in 1 mL of sterile PBS or THB with 10 mg/mL penicillin, homogenized, and passed through a 0.22 µm filter. Samples were frozen at -80 ˚C or on dry ice until analyses were performed. Samples were analyzed by Eve Technologies via multiplex cytokine array (Eve Technologies, Alberta, Canada) as previously described.[8] We have previously validated host targets for specific cytokines (IL-1β, IL-6, KC, and TNF-α) by sandwich ELISA (AbCam).[8, 9]

Statistical analysis. All in vitro data shown signify three independent experiments with two or three technical replicates. Data are expressed as the mean ± SEM. Statistical significance was determined using one-way or two-way analysis of variance (ANOVA) with post hoc Dunnett's multiple-comparisons test or a Student's t test. P values ≤0.05 were determined significant. Effect size (Cohen's D) was calculated from observed differences between the uninfected and infected groups to determine the smallest group size that would enable detection of a significant effect at a minimum desired power of 80%, as previously described.[5] The power statistics were calculated based on the hypothesis focused on proinflammatory cytokines where an effect was expected; namely, IL-6, IFN-γ, and IL-1β. To justify the minimum group size, we focused on the smallest possible effect size among the comparisons of interest, which was IFN-γ. Using the 'pwr' package, a minimum effect size of 1.53 was necessary for power of 80% with a group size of N=3. Cohen's D for the comparison of IFN-γ between groups was 1.67; thus, a minimum group size of 3 individuals is sufficiently powered to detect differences. Statistical analyses were performed in GraphPad Prism Software 10 (GraphPad Prism Software Inc., La Jolla, California) or R (version 4.2.1 in R Studio 2022.07.0.6).

Ethics Statement. This study was carried out in accordance with the recommendations of the Vanderbilt University Medical Center Institutional Review Board. This protocol was approved by the Institutional Review Board (IRB #181998 and #00005756). All animal experiments were performed in S12 accordance with the Animal Welfare Act, U.S. federal law, and NIH guidelines. All experiments were carried out under a protocol approved by Vanderbilt University Institutional Animal Care and Use Committee (IACUC: M/14/034 and M/17/012), a body that has been accredited by the Association of Assessment and Accreditation of Laboratory Animal Care Act (AAALAC).

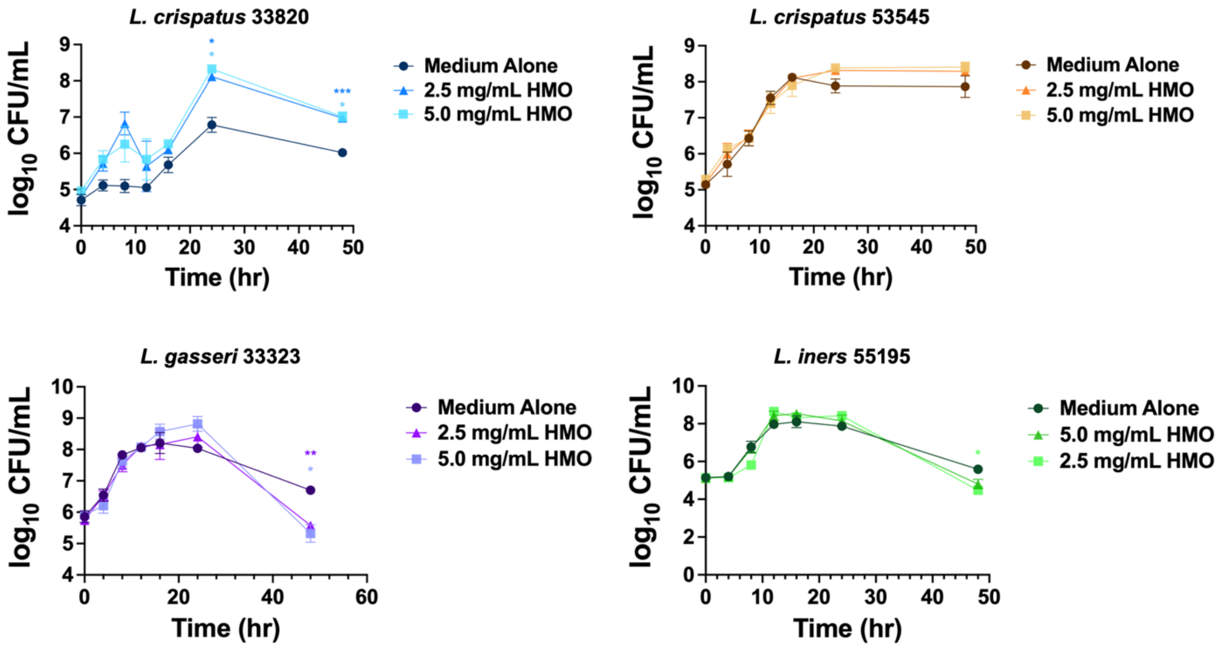

Figure S1. HMOs increase the viability of *L. crispatus* 33820. HMOs (2.5 mg/mL and 5.0 mg/mL) increase the viability of *L. crispatus* 33820, do not impact *L. crispatus* 53545 viability, and decrease viability of *L. gasseri* 33323 and *L. iners* 55195 at their final timepoints. Symbols indicate mean ± SEM. Star color corresponds to the HMO dose used for the comparison. *P<0.05, **P<0.01, ***P<0.001, ****P<0.0001 by two-way ANOVA with Dunnett's post hoc multiple comparisons test (N=3).

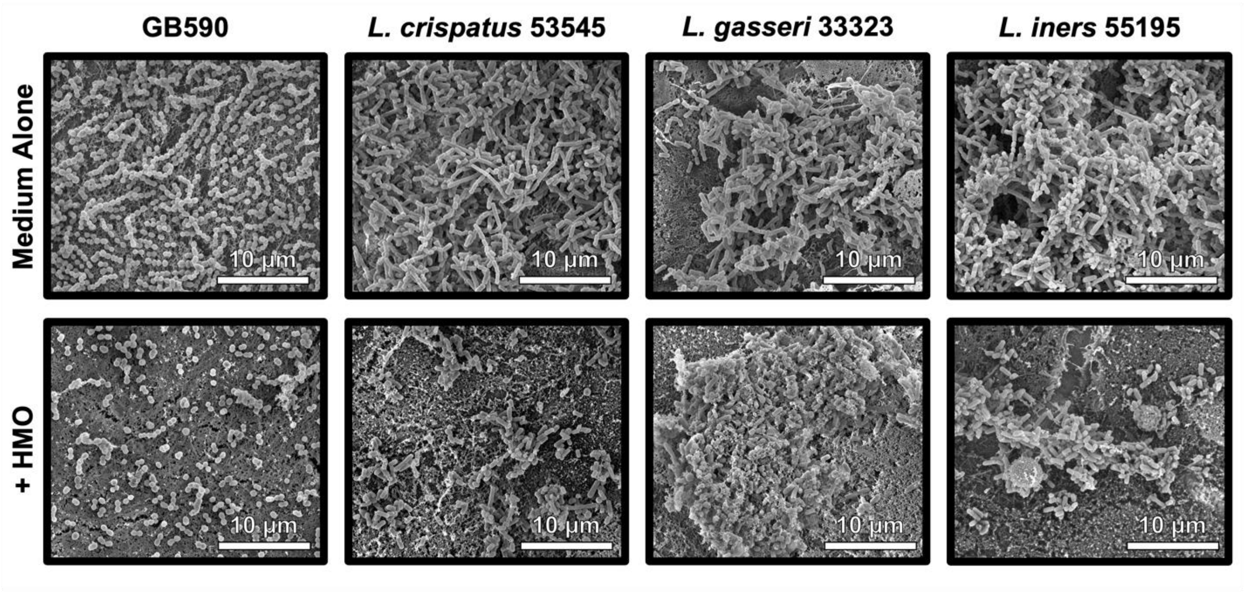

Figure S2. HMOs decrease adherence of all tested bacteria on EpiVaginal tissue. High-resolution field-emission gun scanning electron microscopy (FEG-SEM) analyses of GB590, *L. crispatus* 53545, *L. gasseri* 33323, and *L. iners* 55195 adherence to vaginal tissue. FEG-SEM imaging of bacterial adherence was performed on bacteria grown with vaginal tissues in medium alone (top) or with 10.0 mg/mL of HMOs (bottom). Micrographs were collected at 10,000x magnification, and magnification bars indicate 10 μm.

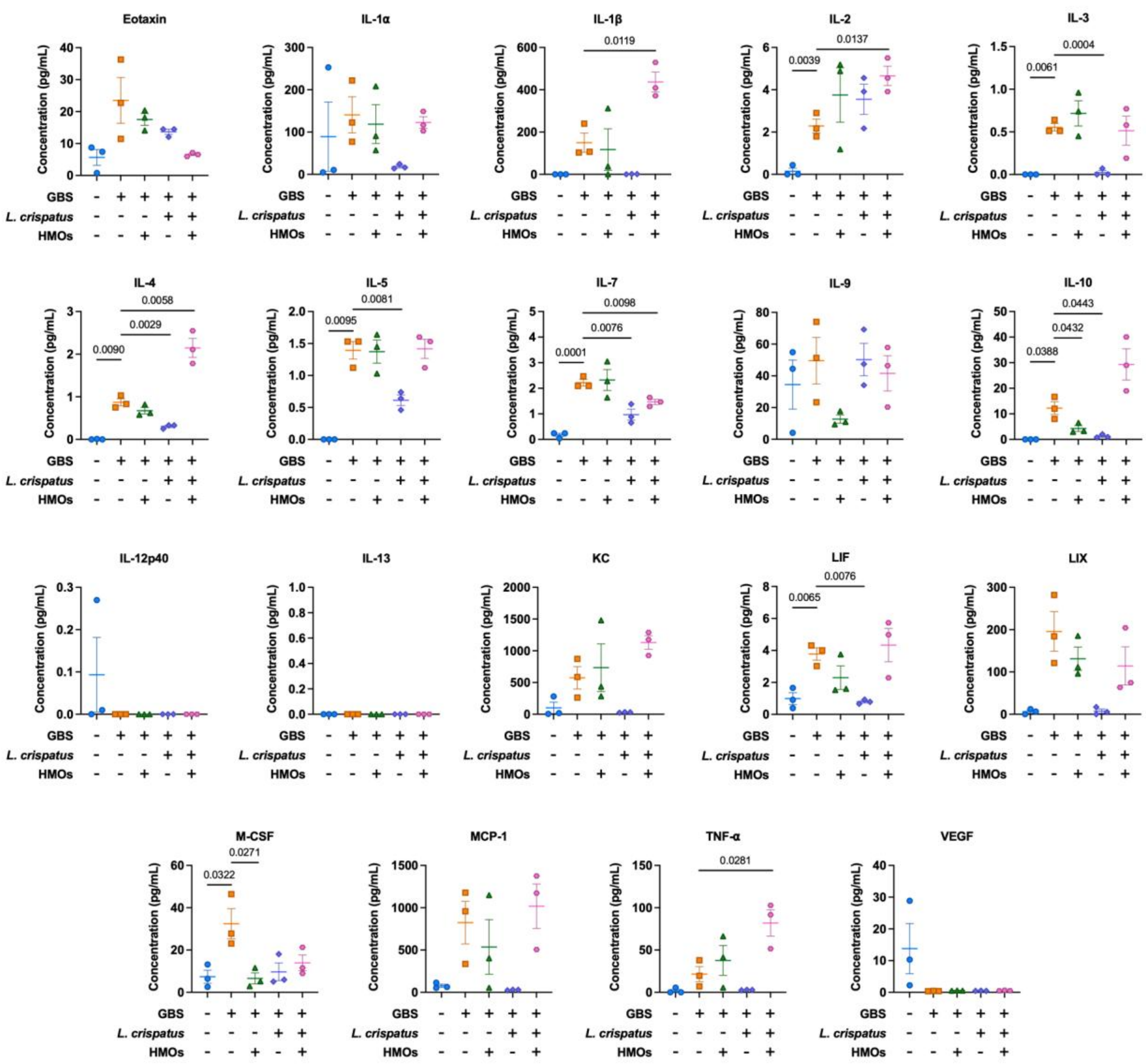

Figure S3. Analysis of cytokine and chemokine production in decidual tissue in response to GBS infection with HMO, *L. crispatus*, or HMO + *L. crispatus* treatment. Multiplex cytokine analyses of decidual tissue after ascending vaginal infection with no infection or treatment (blue circles), GB590 (orange squares), GB590 with HMOs (green triangles), GB590 with *L. crispatus* 33820 (purple diamonds), and GB590 with HMOs and *L. crispatus* 33820 (pink hexagons). Decidual tissues were collected from pregnant dams on E15.5, two days post inoculation. Errors bars represent the standard error mean with individual data points representing analysis of tissue from separate dams. Significance determined via Student's t test, N=3. P values <0.05 were considered significant and those resulting p values are listed on the individual cytokine graphs.

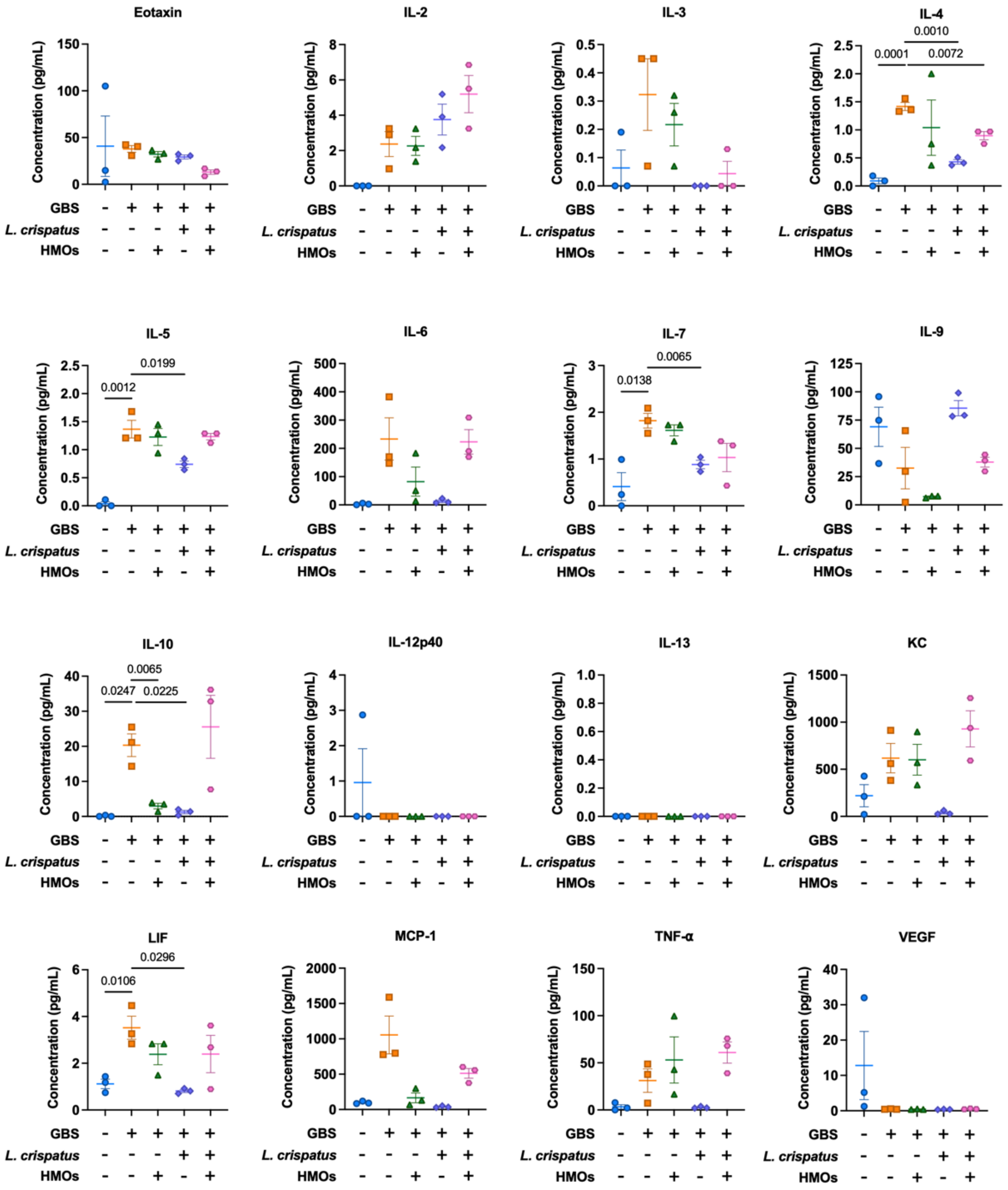

Figure S4. Analysis of cytokine and chemokine production in placental tissue in response to GBS infection with HMO, *L. crispatus*, or HMO + *L. crispatus* treatment. Multiplex cytokine analyses of placental tissue after ascending vaginal infection with no infection or treatment (blue circles), GB590 (orange squares), GB590 with HMOs (green triangles), GB590 with *L. crispatus* 33820 (purple diamonds), and GB590 with HMOs and *L. crispatus* 33820 (pink hexagons). Placental tissues were collected from pregnant dams on E15.5, two days post inoculation. Errors bars represent the standard error mean with individual data points representing analysis of tissue from separate dams. Significance determined via Student's t test, N=3. P values <0.05 were considered significant and those resulting p values are listed on the individual cytokine graphs.

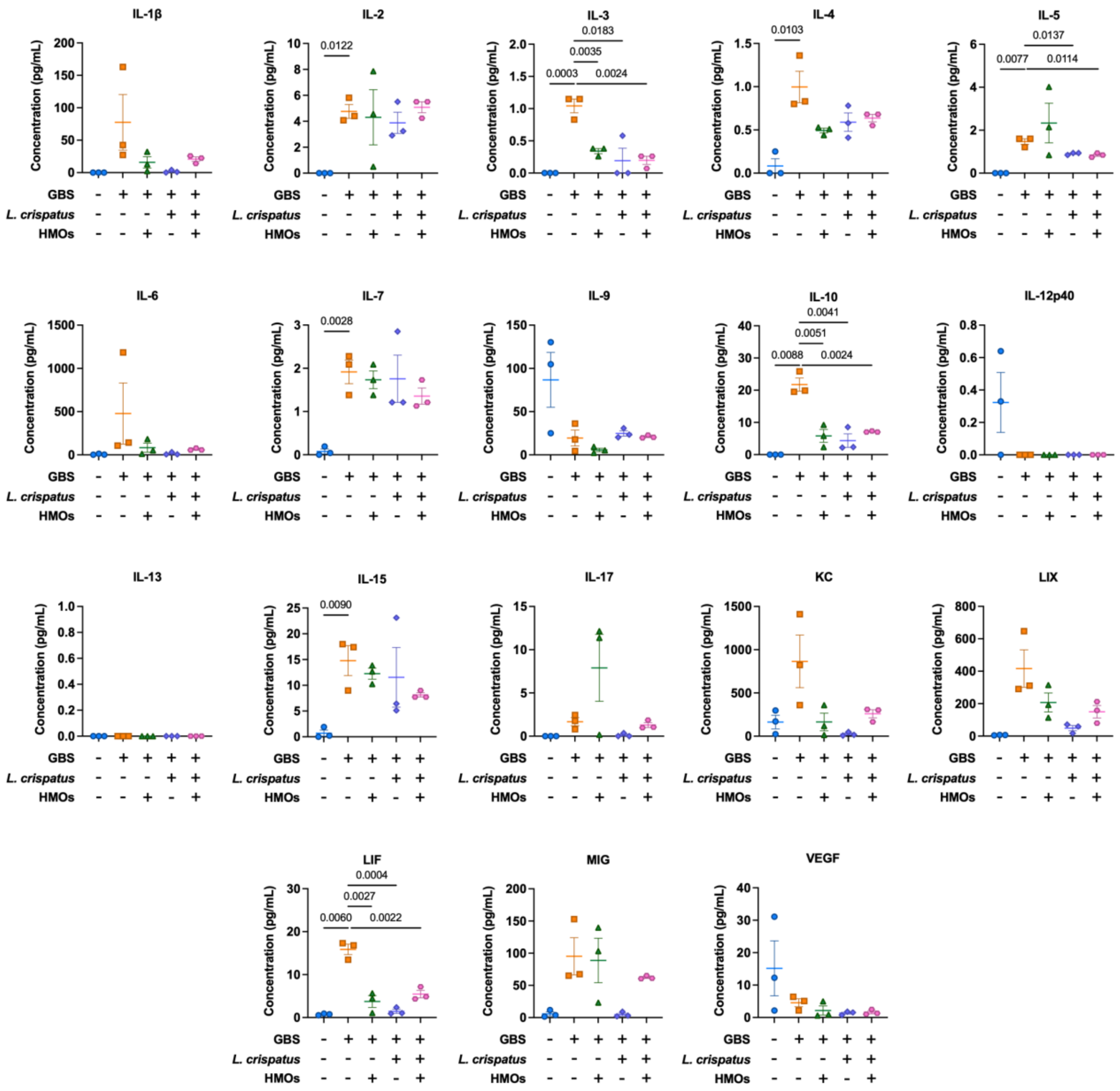

Figure S5. Analysis of cytokine and chemokine production in amnion tissue in response to GBS infection with HMO, *L. crispatus*, or HMO + *L. crispatus* treatment. Multiplex cytokine analyses of amnion tissue after ascending vaginal infection with no infection or treatment (blue circles), GB590 (orange squares), GB590 with HMOs (green triangles), GB590 with *L. crispatus* 33820 (purple diamonds), and GB590 with HMOs and *L. crispatus* 33820 (pink hexagons). Amnion tissues were collected from pregnant dams on E15.5, two days post inoculation. Errors bars represent the standard error mean with individual data points representing analysis of tissue from separate dams. Significance determined via Student's t test, N=3. P values <0.05 were considered significant and those resulting p values are listed on the individual cytokine graphs.

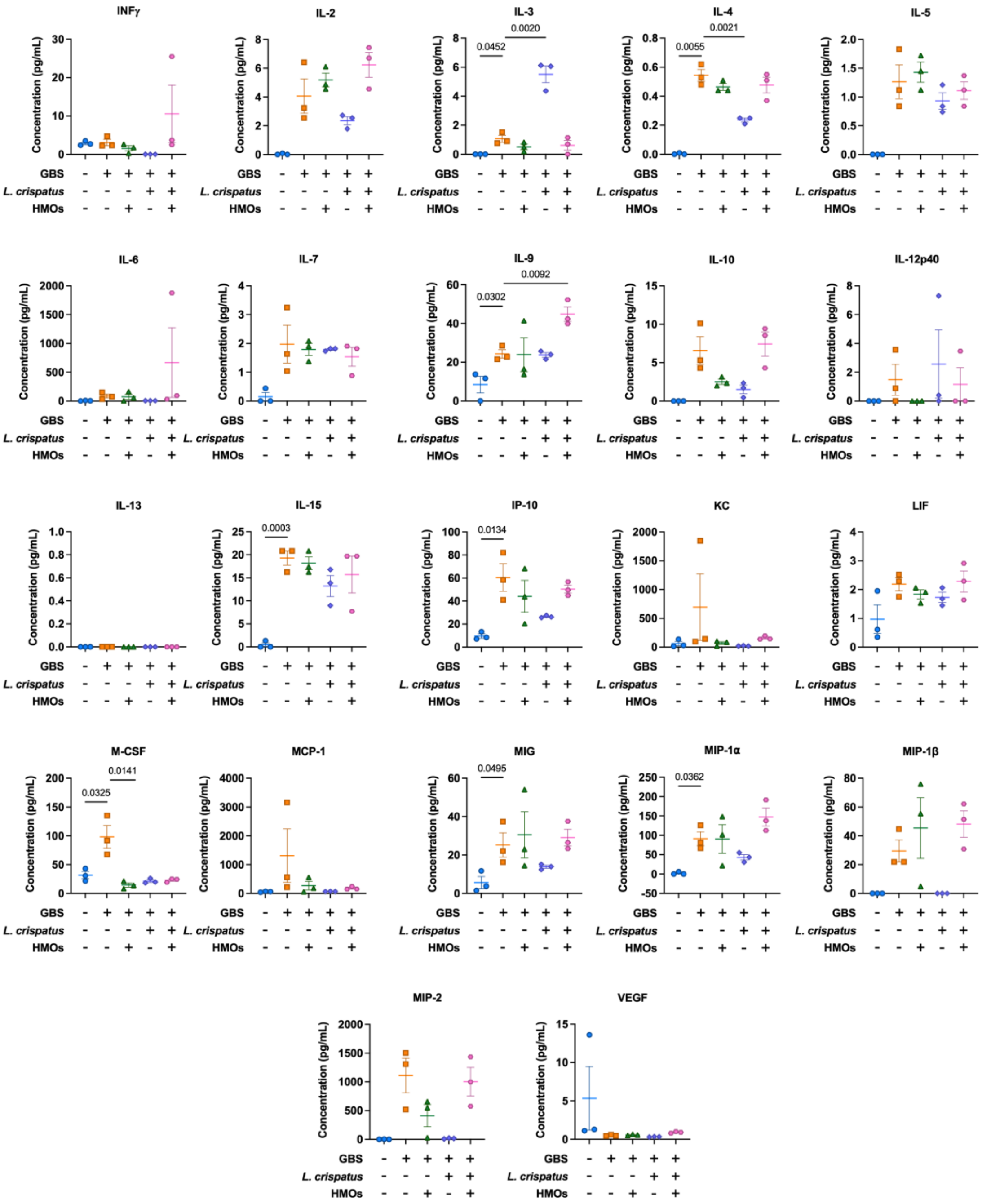

Figure S6. Analysis of cytokine and chemokine production in fetal tissue in response to GBS infection with HMO, *L. crispatus*, or HMO + *L. crispatus* treatment. Multiplex cytokine analyses of fetal tissue after ascending vaginal infection with no infection or treatment (blue circles), GB590 (orange squares), GB590 with HMOs (green triangles), GB590 with *L. crispatus* 33820 (purple diamonds), and GB590 with HMOs and *L. crispatus* 33820 (pink hexagons). Fetal tissues were collected from pregnant dams on E15.5, two days post inoculation. Errors bars represent the standard error mean with individual data points representing analysis of tissue from separate dams. Significance determined via Student's t test, N=3. P values <0.05 were considered significant and those resulting p values are listed on the individual cytokine graphs.

Table S1. Proinflammatory cytokines and chemokines that displayed a significant change in production in response to *L. crispatus* alone or in combination with HMOs during infection.

**Proinflammatory Cytokine/Chemokine**	**Decidua**	**Placenta**	**Amnion**	**Fetus**
Eotaxin			X	X
G-CSF	X	X	X	X
GM-CSF	X	X	X	X
IFN-γ	X	X	X	
IL-1α		X	X	X
IL-1β		X		X
IL-6	X			
IL-12p70	X	X	X	X
IL-15	X	X		
IL-17	X	X		X
IP-10	X	X	X	
KC				
LIX		X		X
MCP-1			X	
MCS-F		X	X	
MIG	X	X		
MIP-1α	X	X	X	
MIP-1β	X	X	X	
MIP-2	X	X	X	
RANTES	X	X	X	X
TNF-α			X	X

References

(1) Shiroda, M.; Aronoff, D. M.; Gaddy, J. A.; Manning, S. D. The impact of *Lactobacillus* on group B streptococcal interactions with cells of the extraplacental membranes. *Microb Pathog* **2020**, *148*, 104463-104463.

(2) Bloom, S. M.; Mafunda, N. A.; Woolston, B. M.; Hayward, M. R.; Frempong, J. F.; Abai, A. B.; Xu, J.; Mitchell, A. J.; Westergaard, X.; Hussain, F. A.; et al. Cysteine dependence of *Lactobacillus iners* is a potential therapeutic target for vaginal microbiota modulation. *Nat Microbiol* **2022**, *7* (3), 434-450.

(3) Manning, S. D.; Lewis, M. A.; Springman, A. C.; Lehotzky, E.; Whittam, T. S.; Davies, H. D. Genotypic diversity and serotype distribution of group B *Streptococcus* isolated from women before and after delivery. *Clin Infect Dis* **2008**, *46* (12), 1829-1837.

(4) Moore, R. E.; Thomas, H. C.; Manning, S. D.; Gaddy, J. A.; Townsend, S. D. Galacto-Oligosaccharide Supplementation Modulates Pathogen-Commensal Competition between *Streptococcus agalactiae* and *Streptococcus salivarius*. *ChemBioChem* **2022**, *23* (3), e202100559.

(5) Moore, R. E.; Spicer, S. K.; Lu, J.; Chambers, S. A.; Noble, K. N.; Lochner, J.; Christofferson, R. C.; Vasco, K. A.; Manning, S. D.; Townsend, S. D.; Gaddy, J. A. The Utility of Human Milk Oligosaccharides against Group B *Streptococcus* Infections of Reproductive Tissues and Cognate Adverse Pregnancy Outcomes. *ACS Cent Sci* **2023**, *9* (9), 1737-1749.

(6) Adams, C. E.; Spicer, S. K.; Gaddy, J. A.; Townsend, S. D. Synthesis of a Phosphoethanolamine Cellulose Mimetic and Evaluation of Its Unanticipated Biofilm Modulating Properties. *ACS Infect Dis* **2024**, *10* (9), 3245-3255.

(7) Kothary, V.; Doster, R. S.; Rogers, L. M.; Kirk, L. A.; Boyd, K. L.; Romano-Keeler, J.; Haley, K. P.; Manning, S. D.; Aronoff, D. M.; Gaddy, J. A. Group B *Streptococcus* Induces Neutrophil Recruitment to Gestational Tissues and Elaboration of Extracellular Traps and Nutritional Immunity. *Front Cell Infect Microbiol* **2017**, *7*.

(8) Doster, R. S.; Kirk, L. A.; Tetz, L. M.; Rogers, L. M.; Aronoff, D. M.; Gaddy, J. A. *Staphylococcus aureus* Infection of Human Gestational Membranes Induces Bacterial Biofilm Formation and Host Production of Cytokines. *J Infect Dis* **2016**, *215* (4), 653-657.

(9) Korir, M. L.; Doster, R. S.; Lu, J.; Guevara, M. A.; Spicer, S. K.; Moore, R. E.; Francis, J. D.; Rogers, L. M.; Haley, K. P.; Blackman, A.; et al. *Streptococcus agalactiae* cadD alleviates metal stress and promotes intracellular survival in macrophages and ascending infection during pregnancy. *Nat Commun* **2022**, *13* (1).
